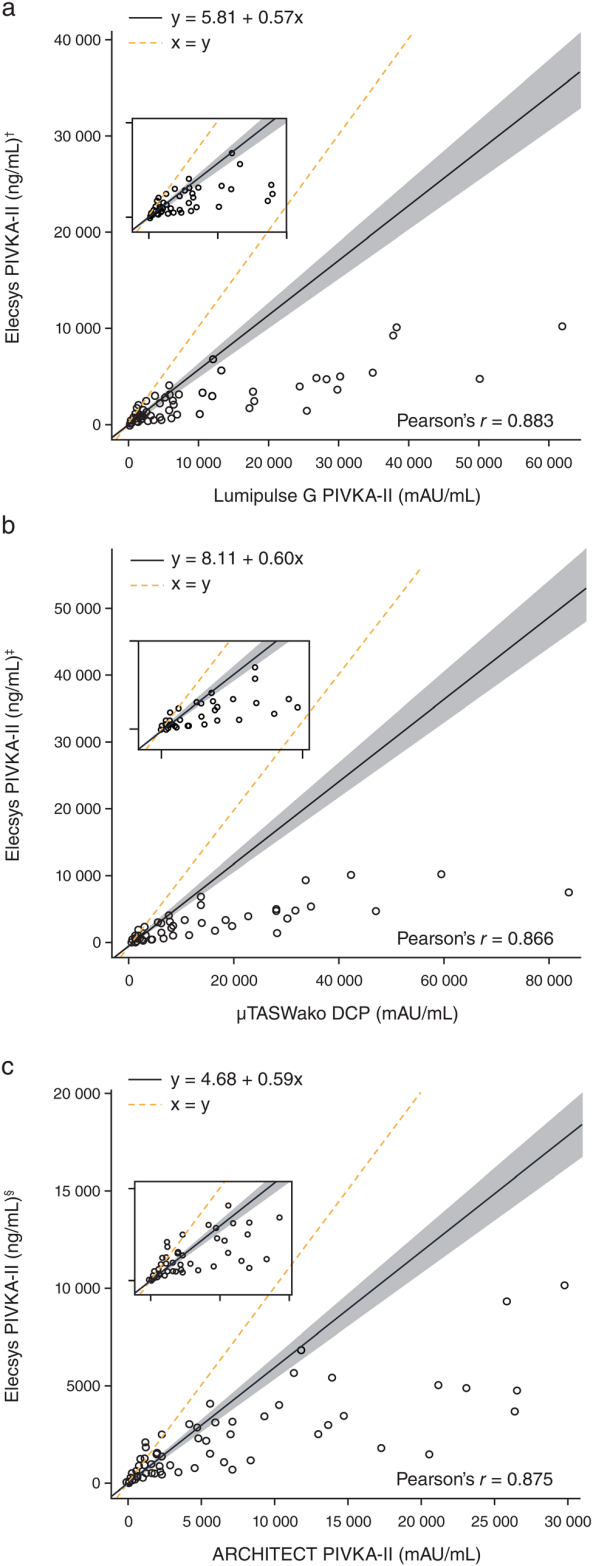# Corrigendum

**DOI:** 10.1002/jgh3.12794

**Published:** 2022-08-02

Henry L Y Chan, Arndt Vogel, Thomas Berg, Enrico N De Toni, Masatoshi Kudo, Jörg Trojan, Anja Eiblmaier, Hanns‐Georg Klein, Johannes Kolja Hegel, Ashish Sharma, Kairat Madin, Vinzent Rolny, Marcus‐Rene Lisy and Teerha Piratvisuth. **Performance evaluation of the Elecsys PIVKA‐II and Elecsys AFP assays for hepatocellular carcinoma diagnosis.** *JGH Open*. 2022; **5:** 292–300.

The publisher wishes to bring the readers’ attention to errors in the above article.

In the article cited above, y values in Figure 1a‐1c were incorrectly displayed as, ‘y = 5.81x + 0.57 Lumipulse G PIVKA‐II’, ‘y = 8.11x + 0.60 μTASWako DCP’, and ‘y = 4.68x + 0.59 ARCHITECT PIVKA‐II’, respectively. The y values should have been, ‘y = 5.81 + 0.57x’, ‘y= 8.11 + 0.60x’, and ‘y = 4.68 + 0.59x’, respectively. The corrected version of Figure 1 is shown in the next page.

In addition, y values in the ‘Results’, ‘Analytical performance’ section were incorrectly displayed as:

**Results**

***Analytical performance*.** […] Weighted Deming regression analyses showed moderate agreement between the Elecsys PIVKA‐II assay and Lumipulse G PIVKA‐II (y = 5.81x + 0.57; Pearson's r = 0.883; P < 0.001); μTASWako DCP (y = 8.11x + 0.60; Pearson's r = 0.866; P < 0.001); and ARCHITECT PIVKA‐II (y = 4.68x + 0.59; Pearson's r = 0.875; P < 0.001) assays (Fig. 1).

The correct sentence should read:

**Results**

***Analytical performance*.** […] Weighted Deming regression analyses showed moderate agreement between the Elecsys PIVKA‐II assay and Lumipulse G PIVKA‐II (y = 5.81 + 0.57x; Pearson's *r* = 0.883; *P* < 0.001); *μ*TASWako DCP (y = 8.11 + 0.60x; Pearson's *r* = 0.866; *P* < 0.001); and ARCHITECT PIVKA‐II (y = 4.68 + 0.59x; Pearson's *r* = 0.875; *P* < 0.001) assays (Fig. 1).

The online version of this article was corrected.

We would like to apologize for these errors and any confusion they may have caused.